# A Large Language Model Workflow for Auditable Brain Abscess Risk Stratification and Pre-residency Scholarship: A Technical Report

**DOI:** 10.7759/cureus.100415

**Published:** 2025-12-30

**Authors:** Amir Akhavan, Swapan Nath

**Affiliations:** 1 Medicine, Anne Burnett Marion School of Medicine at Texas Christian University, Fort Worth, USA; 2 Medical Education, Anne Burnett Marion School of Medicine at Texas Christian University, Fort Worth, USA

**Keywords:** artificial intelligence, brain abscess, evidence synthesis, large language models, medical education, mentorship, prompt engineering, risk stratification, undergraduate medical education

## Abstract

Large language models (LLMs) are increasingly available to medical trainees, but transparent and auditable methods for incorporating them into scholarly work and emerging artificial intelligence (AI) literacy curricula remain limited. This technical report describes a mentored educational framework in which a de-identified brain abscess case report was transformed into a reproducible, LLM-supported risk-stratification model to teach rigor, verification, and structured AI use to a fourth-year medical student. A single clinical case was reconstructed using structured variables from history, imaging, laboratory trends, and symptom trajectory. Each interaction with the LLM followed a standardized query pattern and was logged in a prompt ledger capturing prompts, rationales, and inclusion or exclusion decisions; chain-of-thought outputs were retained only as reasoning traces for supervised review. Literature-supported deterioration indicators were synthesized into a provisional Neurologic Deterioration in Brain Abscess Score (NDBAS v0.1), emphasizing lesion diameter, midline shift, vasogenic edema, inflammatory markers, sensorium changes, and metabolic risk factors. Applying NDBAS v0.1 to the index case yielded a score of 5, corresponding to a moderate-risk tier. Relative to the learner’s prior manual approach, the LLM-assisted synthesis accelerated evidence review while maintaining rigor through primary-source verification and faculty oversight and produced three concrete curriculum artifacts: a narrative case appendix, a prompt ledger, and a variable dictionary with domain-specific cut points and tiered risk thresholds. This structured, audit-ready LLM workflow offers a practical route to building pre-residency AI literacy by embedding a supervised “Prompt Ledger plus ask-verify-revise” pattern into complex case-based learning, moving students from casual chatbot use to transparent evidence synthesis, critical appraisal, and conceptual model-building from single cases. With appropriate governance, de-identification, and faculty oversight, similar mentored workflows can help learners at other institutions turn single cases into auditable conceptual models, strengthening both their scholarly output and their readiness to engage with AI responsibly during clinical training.

## Introduction

Large language models (LLMs) are entering clinical and educational workflows, and medical students are increasingly encountering these tools in study and scholarship. ChatGPT has performed at or near the passing threshold for United States Medical Licensing Examination (USMLE)-style questions, suggesting potential value for AI-assisted learning when used appropriately [1]. In parallel, national and international bodies are calling for structured, transparent use of artificial intelligence (AI) in health professions education, emphasizing governance, ethics, and deliberate integration into curricula rather than informal, unsupervised use [2,3]. Within this landscape, the primary contribution of this technical report is not a new risk score per se, but a reproducible, faculty-supervised workflow that medical schools can adapt to teach responsible LLM use in pre-residency scholarship.

Brain abscess provides a deliberately demanding test case for such a workflow. It is an uncommon but high-stakes diagnosis in which deterioration signals emerge across neuroimaging, laboratory trends, and the clinical narrative, and integrating these signals can be challenging for trainees [4,5]. In this project, a de-identified case of a 60-year-old male with diabetes and poor dentition who presented as a stroke mimic and was diagnosed with a left hemispheric brain abscess was reconstructed into a structured dataset. Under faculty supervision, a fourth-year medical student used an LLM to surface literature-supported predictors of neurologic deterioration and assemble them into a conceptual Neurologic Deterioration in Brain Abscess Score (NDBAS v0.1). The score is explicitly framed as an educational prototype rather than a validated clinical decision tool; the focus is on how the model is used, not on bedside performance.

Because LLMs can hallucinate and obscure their reasoning, supervised scholarly use requires explicit guardrails. Emerging AI reporting and governance frameworks, such as the Consolidated Standards of Reporting Trials-Artificial Intelligence (CONSORT-AI) extension, the Reporting Guideline for the Early-Stage Clinical Evaluation of Decision Support Systems Driven by Artificial Intelligence (DECIDE-AI), and the Minimum Information about Clinical Artificial Intelligence Modeling (MI-CLAIM) checklist, stress clear documentation of AI inputs, outputs, and human-AI interactions, along with reproducible methods and human oversight [3,6-8]. In our workflow, every LLM interaction was captured in a prompt ledger, all candidate variables were verified against primary literature, and only de-identified case data were used. These practices align with ethical AI guidance from the World Health Organization and with calls from academic medicine to prioritize rigor, reproducibility, and transparency in AI-enabled work [2,3,6-8].

Pedagogically, the project sits at the intersection of cognitive apprenticeship and self-regulated learning. Cognitive apprenticeship emphasizes that learners benefit when experts make their reasoning visible, coach performance, and gradually fade support as competence develops [9]. Self-regulated learning frameworks similarly highlight planning, monitoring, and reflecting on tasks as students work toward independent mastery [10]. Here, the mentor and student jointly framed clinical questions, evaluated LLM outputs, and discussed uncertainties, turning each interaction into practice in goal setting, monitoring, and reflection rather than passive answer-seeking. Prior work has already shown that LLMs can approach passing thresholds on high-stakes exams and encode substantial clinical knowledge [1,11], and we previously demonstrated a similar mentored pattern in a pulmonary infection risk-stratification project [12]. The present report extends that work into a central nervous system infection and stroke-mimic context, foregrounding an auditable, supervised workflow as the main product.

On the AI side, we used structured prompts to make the model’s reasoning explicit while keeping humans in control. Chain-of-thought prompting, asking the model to break its reasoning into intermediate steps, has been shown to improve performance on complex problems [13]. Zero-shot chain-of-thought prompting demonstrates that even simple cues such as “Let’s think step by step” can elicit multi-step reasoning without worked examples [14]. In our workflow, such outputs were treated not as ground truth but as auditable traces to be inspected, critiqued, and, when necessary, discarded. Overall, this technical report centers on a supervised, audit-ready interaction pattern in which LLMs function as transparency aids for medical student scholarships, rather than as opaque oracles.

This paper distinguishes our contribution and provides a replicable educational model for safe, effective integration of LLM-assisted synthesis into medical training. It also builds on earlier LLM-assisted risk-stratification work in a pulmonary infection case report [12], extending that pattern into a CNS infection, stroke-mimic scenario.

The work was presented locally at the 2025 Faculty Research Symposium at the Anne Burnett Marion School of Medicine at Texas Christian University (October 21, 2025) and nationally at IDWeek 2025 (Infectious Diseases Society of America) in Atlanta, Georgia (October 20, 2025; Abstract ID: 2114961).

## Technical report

Case and data compilation

We used a detailed clinical case of a 60-year-old male with a brain abscess, incorporating the full narrative history (epidemiology), risk factors, imaging interpretations, laboratory data, and progression/severity trajectory. The complete narrative case report is provided in the Appendix for the reader's context. For the technical workflow, the student reconstructed this narrative into a structured, time-stamped dataset to preserve relationships among presentation, neuroimaging findings, and laboratory trends.

The patient awoke with right-sided weakness, dysarthria, and left facial droop, initially raising concern for stroke. Non-contrast CT demonstrated ill-defined edema, and MRI revealed a bilobed, rim-enhancing lesion measuring approximately 2.6 cm with associated vasogenic edema and mild rightward midline shift. Admission leukocytosis suggested systemic inflammatory activity over time, consistent with prior descriptions in brain abscess cohorts [4,5]. Poor dentition raised concern for an odontogenic (endogenous) source of infection [4].

All clinical fields were fully de-identified at capture. Names, medical record numbers, dates, addresses, and institutional identifiers were removed, and event timing was encoded using relative markers such as day 0, day +7, day +14, and day +21. Data were stored in a versioned spreadsheet with one row per observation and columns for timestamp, domain, variable, value, and notes. No DICOM (Digital Imaging and Communications in Medicine) files or radiologic images were supplied to the LLM; only textual radiology summaries and laboratory values were used so that the model analyzed clinician interpretations rather than pixel-level image data.

Before engaging the model, the mentor and MS4 student agreed on specific inquiry domains (history, imaging, laboratory indices, systemic inflammatory markers, and source clues) to identify literature-supported predictors of early neurologic deterioration in brain abscess. The predictors were then translated into a transparent, point-based framework, the Neurologic Deterioration in Brain Abscess Score (NDBAS v0.1), rather than a deployable clinical tool [4,5]. This marked a deliberate transition from a traditional descriptive case report to a structured, AI-assisted workflow intended to promote rigorous evidence synthesis.

LLM-assisted synthesis workflow

The interaction protocol followed a controlled “ask-verify-revise” cycle instead of open-ended conversational use. For each step, the learner posed a short, clinically phrased question anchored to the case and explicitly requested primary literature support. Each query contained: (1) a ≤5-line synopsis of the de-identified case (for context); (2) a single domain-scoped clinical question; and (3) a direct request for citations. A representative prompt was as follows: “Given a 60-year-old man with a ~2.6 cm ring-enhancing lesion and mild midline shift, which imaging features most strongly predict early neurologic deterioration in brain abscess? Cite primary sources.” Other prompts asked the model to summarize predictors of complications across demographics, imaging features (size, rim enhancement, restricted diffusion, edema, midline shift), laboratory trends (white blood cell count, C-reactive protein), and source clues such as poor dentition, then to map those predictors back onto the index case [4,5,11]. Representative prompts, purposes, and validation checkpoints are summarized in Table 1.

**Table 1:** Representative mentored‑LLM prompts (domain‑scoped) with purposes and validation checkpoints. Abbreviations: LLM, large language model; CoT, chain of thought; WBC, white blood cell count; CRP, C‑reactive protein; NDBAS, Neurologic Deterioration in Brain Abscess Score; DOIs: digital object identifiers; PMIDs: PubMed identifiers; R2T, rigor, reproducibility, and transparency.

ID	Domain & exact prompt	Purpose in workflow	Validation checkpoint
P1	Initial literature scan (no case data): “What clinical parameters are reported in the literature as predictors of complications or poor outcome in brain abscess?”	Establish a canonical, case-agnostic predictor set to reduce anchoring.	Cross-check candidate predictors against primary reviews and radiology/neurosurgery criteria; discard items without primary support.
P2	Structured domains query: “Summarize predictors focusing on: (a) demographics/history; (b) CT/MRI features (size, rim enhancement, restricted diffusion, edema, midline shift); (c) laboratory indices (WBC, CRP); (d) source clues (e.g., poor dentition).”	Force comprehensive retrieval across bedside domains mirrored in the case template.	Require ≥2-3 independent primary sources per candidate predictor; record DOIs/PMIDs in the ledger.
P3	Contextualized case query: “Given a ~2.6 cm rim-enhancing lesion with vasogenic edema and mild rightward midline shift, focal deficits, leukocytosis, and poor dentition, which red-flag features align with known predictors?”	Map literature-based predictors to the index patient; identify immediate risks.	Verify each mapped item against the de-identified imaging reports/lab timeline; note any uncertainties for mentor review.
P4	Scoring/hypothesis prompt: “Propose a point-based scheme integrating these parameters to estimate early neurologic deterioration.”	Generate a provisional weighting sketch (NDBAS v0.1) for mentor adjudication.	Reject opaque weights; retain only weights justifiable by pathophysiology or recurrent multi-study signals.
P5	Citation surfacing: “List the primary studies relied upon for each included predictor and note any conflicting evidence.”	Ensure traceability, surface conflicts, and support R2T audit.	Spot-check citations; flag contradictions and “park” for cohort testing; document keep/modify/discard decisions.
P6	Zero-shot CoT check: “Let’s reason step by step: which combinations of size, midline shift, and changes in sensorium best predict early deterioration in brain abscess?”	Elicit decomposed reasoning for coaching/error-finding.	Treat CoT as an auditable trace only; compare steps to primary sources before adopting any conclusion.

After each model response, the student and mentor verified suggested citations against primary sources, discarded low-yield or irrelevant tangents (for example, fungal abscess pathways not applicable to this case), and tightened or re-ran prompts when answers were vague or insufficiently supported. Predictors that recurred across independent primary studies and aligned with known pathophysiology were flagged for inclusion in NDBAS, with particular emphasis on lesion diameter and midline shift because of their prominence in radiologic severity indices and prognostic scoring [4,5].

Zero-shot chain-of-thought prompting was used selectively when multi-factor patterns needed to be decomposed into intermediate reasoning steps; for example, asking the model to “think step by step” about how combinations of size, midline shift, and sensorium changes influence risk. These chain-of-thought texts were treated strictly as auditable reasoning traces rather than ground truth and were reviewed by the mentor for error-finding and coaching, consistent with prior work on chain-of-thought and zero-shot reasoning in LLMs [13,14].

Audit artifacts: prompt ledger and variable dictionary

To make the workflow auditable and reproducible, we relied on two linked artifacts. The first was the prompt ledger (Table 2), which recorded for each interaction the exact prompt text, model identifier, timestamp, case context, surfaced references (with digital object identifiers (DOIs) or PubMed IDs when available), and the mentor’s decision to keep, modify, or discard the output.

**Table 2:** Prompt ledger. The prompt ledger was maintained to record each mentored large language model (LLM) exchange and its purpose, along with subsequent validation steps. For each run, the prompt ledger stored the prompt text, model identifier, timestamp, relevant case snippet, references surfaced (with DOIs or PubMed IDs), and the mentor’s decision to keep, modify, or discard the proposed variables and rationales. All interactions were logged chronologically, allowing future educators to reconstruct the analytic process step by step. Abbreviations: NDBAS, Neurologic Deterioration in Brain Abscess Score; DOIs: digital object identifiers.

Iteration	Session/week	Timestamp (relative)	Model	Domain/case context included	Exact prompt	Returned key factors (summary)	Citations surfaced	Mentor decision	Rationale/notes
1	Week 1/Session A	Day 0, 09:15	ChatGPT-4o3 Deep Research	General (no case data); case context included: No	What clinical parameters are reported in the literature as predictors of complications or poor outcomes in brain abscesses?	Abscess diameter; midline shift; level of consciousness (Glasgow Coma Scale, GCS); edema; multiplicity/location; inflammatory markers; host factors (diabetes, immunosuppression).	Brouwer 2014 N Engl J Med; Demir 2007 Clin Radiol	Keep (as canonical set)	Establish case-agnostic baseline predictors to prevent anchoring before seeing the index case.
2	Week 1/Session B	Day 0, 10:05	ChatGPT-4o3 Deep Research	Structured domains (demographics/history; CT/MRI features; labs; source clues); case context included: No	Focusing on (a) demographics/history; (b) CT/MRI features (size, rim enhancement, restricted diffusion, edema, midline shift); (c) laboratory indices (white blood cell count, C-reactive protein); and (d) source clues (e.g., poor dentition), summarize which parameters are most strongly associated with complications in brain abscess.	Confirms Iteration 1; adds that mass-effect indicators (size around 3.0 cm and shift around 5 mm) carry a higher risk and often necessitate surgery.	Brouwer 2014 N Engl J Med; Demir 2007 Clin Radiol	Keep	Converges with Iteration 1 and provides thresholds to test.
3	Week 1/Session C	Day 0, 11:20	ChatGPT-4o3 Deep Research	Case-context mapping; case context included: Yes	Given a ~2.6 cm rim-enhancing lesion with vasogenic edema and mild rightward midline shift, focal deficits, leukocytosis, and poor dentition, which red-flag features align with known predictors of early neurologic deterioration?	Size near 2.6 cm; presence of edema; measurable (though mild) midline shift; leukocytosis; diabetes as a comorbidity; probable odontogenic source.	Brouwer 2014 N Engl J Med; Demir 2007 Clin Radiol	Keep (map to index case)	Maps literature-based predictors to the patient; confirms several risk elements are present.
4	Week 1/Session D	Day 0, 12:10	ChatGPT-4o3 Deep Research	Scoring/hypothesis; case context included: Yes	Propose a point-based scheme integrating these parameters to estimate early neurologic deterioration in brain abscess.	Suggests ordinal weighting: size, midline shift, and GCS as primary drivers; edema, inflammatory trend, and comorbidity as modifiers.	Brouwer 2014 N Engl J Med; Demir 2007 Clin Radiol (used as anchors)	Modify	Reject opaque weights; adopt a conservative 0-2 scale and require explicit thresholds with literature anchors.
5	Week 2/Session A	Day +7, 09:00	ChatGPT-4o3 Deep Research	Citation surfacing; case context included: No	List the primary studies relied upon for each included predictor and note any conflicting evidence.	Confirms radiology severity-index support for size and shift; notes variability in C-reactive protein utility; emphasizes sensorium as a key clinical correlate.	Brouwer 2014 N Engl J Med; Demir 2007 Clin Radiol	Keep (with flags)	C-reactive protein threshold flagged as uncertain and parked for cohort testing; other predictors retained.
6	Week 2/Session B	Day +7, 10:30	ChatGPT-4o3 Deep Research	Zero-shot chain-of-thought check; case context included: Yes	Reason step by step: Which combinations of size, midline shift, and changes in sensorium best predict early deterioration in brain abscess?	Highlights interaction of size in the 2.5–3.0 cm or larger range, plus midline shift >=5 mm with any GCS decline as a high-risk phenotype.	Brouwer 2014 N Engl J Med; Demir 2007 Clin Radiol	Keep (trace only)	Treat the chain-of-thought as an auditable trace; accept the conclusion only insofar as it aligns with primary sources.
7	Week 3/Session A	Day +14, 09:20	ChatGPT-4o3 Deep Research	Refinement of thresholds; case context included: No	Provide explicit thresholds for midline shift and abscess diameter, with citations, that could be used in a scoring scale.	Diameter: <2.5 cm = 0, 2.5–3.0 cm = 1, >3.0 cm = 2; midline shift: none = 0, 1-4 mm = 1, >=5 mm = 2.	Demir 2007 Clin Radiol	Keep	Thresholds adopted for NDBAS v0.1; to be stress-tested in a cohort.
8	Week 3/Session B	Day +14, 10:10	ChatGPT-4o3 Deep Research	Refinement of laboratory trends; case context included: No	Summarize evidence for C-reactive protein and white blood cell count trends as independent predictors of worsening in brain abscess.	White blood cell rise often accompanies uncontrolled infection; the C-reactive protein signal is inconsistent; limited quantitative thresholds are available.	Brouwer 2014 N Engl J Med (review synthesis)	Modify	Retain inflammatory markers as modifiers (0-1); avoid hard thresholds until cohort data are available.
9	Week 4/Session A	Day +21, 09:05	ChatGPT-4o3 Deep Research	Schema lock; case context included: Yes	Using the provisional thresholds, compute the index case’s score and list which variables drive the risk tier.	Score = 5 (moderate). Contributors: size (1), midline shift (1), edema (1), inflammatory activity (1), diabetes (1).	Demir 2007 Clin Radiol; Brouwer 2014 N Engl J Med	Keep	Concordant with the clinical decision for urgent drainage; schema ready for cohort testing.

From an educational standpoint, the prompt ledger documented not only what the model produced but how the student interacted with it: which prompts were effective, where chains of thought contained errors, which suggestions were pruned as tangential, and when additional literature searches were required. This created an assessment artifact that allowed direct observation of the learner’s developing skills in question formulation, evidence appraisal, and threshold selection, consistent with cognitive apprenticeship and self-regulated learning frameworks [9,10]. Weekly mentor-student debriefs used this ledger to refine thresholds, resolve disagreements, and park unresolved questions for future cohort testing, converting otherwise ephemeral chat history into a stable, assessable work product consistent with cognitive apprenticeship and self-regulated learning principles [9,10]. The requirement to defend each variable and cut point with primary citations shifted the activity from passive consumption of AI output to active construction of a defensible variable dictionary and conceptual risk stratification model [3,6-8,11,12]. Once candidate predictors stabilized, they were organized by domain (sensorium, imaging, laboratory, history/comorbidity, and source or pathogen). The second artifact was the variable dictionary for NDBAS v0.1 (Table 3).

**Table 3:** Variable dictionary. All content in the variable dictionary is fully de-identified, and NDBAS v0.1 is clearly labeled as an educational and conceptual prototype rather than a validated clinical decision tool. The variable dictionary and scoring rules are detailed here, which specify for each predictor the operational definition, encoding, thresholds, and provisional weight and flags how missing data are managed, in line with transparency expectations for clinical AI models. Abbreviations: GCS, Glasgow Coma Scale; SAG, Streptococcus anginosus group; DWI, diffusion-weighted imaging.

Domain	Variable	Definition	Units/encoding	Measurement source	Data type	Allowed values/thresholds	Provisional weight (0-2)	Scoring rule (pseudo-code)	Evidence anchor (refs)	Notes/assumptions	Index-case value
Sensorium	Glasgow Coma Scale (GCS)	Best GCS at presentation (pre-sedation).	Integer 3-15	ED/initial neurologic examination note	Integer	0: 15; 1: 13-14; 2: <=12	0/1/2	Score = 0 if GCS = 15; 1 if 13 <= GCS <= 14; 2 if GCS <= 12	Brouwer 2014; clinical severity correlates	The lowest value within the first 6 hours is preferred if fluctuating.	15
Imaging	Abscess diameter (largest)	Maximal diameter of dominant rim-enhancing lesion.	cm (one decimal)	MRI report (axial) or CT if MRI unavailable	Float	0: <2.5; 1: 2.5-3.0; 2: >3.0	0/1/2	Score = 2 if d > 3.0; 1 if 2.5 <= d <= 3.0; else 0	Brouwer 2014; Demir 2007 (severity index)	If multiple lesions, use the largest for this item; multiplicity captured separately.	~2.6
Imaging	Midline shift	Maximum displacement of the septum pellucidum or pineal relative to midline.	mm	CT/MRI report	Integer	0: none; 1: 1-4 mm; 2: >=5 mm	0/1/2	Score = 2 if shift >= 5; 1 if 1 <= shift <= 4; 0 if shift = 0	Demir 2007 (mass-effect index)	Use the largest reported value within a 24-hour window.	~2 mm
Imaging	Perilesional edema	Qualitative extent of vasogenic edema around the dominant lesion.	Ordinal: none/mild, moderate, severe	MRI/CT report narrative	Categorical (ordinal)	0: none/mild; 1: moderate; 2: severe	0/1/2	Score = 0 for none/mild; 1 for moderate; 2 for severe	Brouwer 2014	If ambiguous, default to the lower category and flag “uncertain”.	Moderate
Imaging	Number/location	Multiplicity and eloquence of the involved region.	Categorical	MRI/CT report	Categorical (ordinal)	0: single, non-eloquent region; 1: multiple or eloquent regions; 2: posterior fossa plus multiple lesions	0/1/2	Score = 2 if posterior_fossa AND multiple; else if multiple OR eloquent: 1; else 0	Brouwer 2014	“Eloquent” = primary motor, language, brainstem, or cerebellar outflow; refine in cohort.	Single, supratentorial (frontoparietal)
Imaging (exploratory)	Restricted diffusion (DWI)	Presence of diffusion restriction within the ring-enhancing lesion.	Yes/No	MRI report	Boolean	Not scored in v0.1	—	—	Brouwer 2014 (diagnostic)	Kept for descriptive completeness; may inform differential vs. necrotic tumor.	Present
Laboratory	WBC count (trend)	Leukocyte count and 48-72 hour trajectory on therapy.	cells/µL; trend: rising/stable/falling	Laboratory information system	Numeric + categorical	0: normal or stable; 1: leukocytosis or rising; 2: escalating on appropriate therapy	0/1/2 (modifier)	Score = 2 if rising_on_therapy; 1 if leukocytosis_or_rising; else 0	Brouwer 2014 (review synthesis)	Define “escalating” as >=20% rise over 48-72 hours after therapy initiation.	Leukocytosis present
Laboratory	C-reactive protein (CRP) trend	CRP baseline and trajectory.	mg/L; trend: rising/stable/falling	Laboratory information system	Numeric + categorical	0: stable or declining; 1: rising; 2: escalating after therapy	0/1/2 (modifier)	Score = 2 if escalating_post_therapy; 1 if rising; else 0	Brouwer 2014 (non-specific)	Signal inconsistent; retained as a modifier pending cohort validation.	Not available
Laboratory (informational)	Procalcitonin	Serum procalcitonin level at presentation.	ng/mL	Laboratory information system	Numeric	Not scored in v0.1	—	—	Brouwer 2014 (may be low in localized CNS infection)	Captured for completeness; not a reliable discriminator for localized abscess.	Not measured
History/comorbidity	Comorbidity	The highest-risk host factor present at baseline.	Categorical: none; diabetes/vascular risk; immunosuppression	History and medication list	Categorical (ordinal)	0: none; 1: diabetes or vascular risk; 2: immunosuppression	0/1/2	Score = 2 if immunosuppressed; 1 if diabetes_or_vascular; else 0	Brouwer 2014	Immunosuppression includes steroids >=20 mg prednisone-equivalent for >=2 weeks, chemotherapy, or HIV with CD4 <200.	Diabetes present
Source/pathogen (modifier)	Odontogenic/SAG clue	Clinical or microbiologic evidence of dental source or SAG involvement.	Yes/No	Dental exam; culture if available	Boolean	+1 modifier if confirmed in cohort	+0 (v0.1); reserve +1 pending data	Modifier = 1 if odontogenic_or_SAG_confirmed_and_validated else 0	Brouwer 2014 (etiologic patterns)	Held as non-core until predictive signal confirmed in cohort study.	Poor dentition; SAG likely

For each variable, the dictionary specifies an operational definition, data source, encoding scheme (for example, categorical cut points), allowed values, a provisional 0-2 weight, and a machine-readable scoring rule. Imaging thresholds for abscess diameter and midline shift were anchored to the imaging severity index described by Demir et al. and the clinical syntheses by Brouwer et al. [4,5]. Edema, multiplicity, and lesion location followed standard radiologic descriptors, while inflammatory markers such as elevated white blood cell count and C-reactive protein were treated as modifiers rather than core drivers because of heterogeneous evidence in prior reports [4].

Across nine documented iterations in the ledger, the workflow repeatedly converged on a stable cluster of deterioration signals, including the largest lesion diameter, degree of midline shift, extent of edema, Glasgow Coma Scale, inflammatory activity, and key comorbidities such as diabetes, echoing variables reported in prior prognostic work [4,5]. Six iterations were accepted as-is or with minor edits after citation checks, two were modified because proposed cut points or weights were opaque or weakly supported, and one was retained only as a chain-of-thought trace rather than a source of variables. This pattern demonstrates that the LLM was not treated as a one-step oracle: suggestions were iteratively filtered through primary literature and pathophysiologic plausibility [3,6-8,11]. Variables and thresholds that survived this process were encoded into NDBAS v0.1 with explicit cut points (for example, abscess diameter = <2.5 cm, 2.5-3.0 cm, >3.0 cm; midline shift = none, 1-4 mm, ≥5 mm), mapped to conservative 0-2 weights anchored to prior severity indices [4,5]. The resulting variable dictionary and scoring rules, detailed in Table 3, specify operational definitions, encodings, thresholds, provisional weights, and handling of missing data, consistent with transparency expectations for clinical AI modeling [8]. For replication, three elements are essential: (1) structured and fully de-identified case reconstruction; (2) a domain-scoped ask-verify-revise prompting cycle documented in a prompt ledger; and (3) consolidation of stable predictors into a variable dictionary with explicit cut points and risk tiers. The granular contents of Tables 2, 3 are provided as illustrative examples; educators and researchers may adapt their level of detail to local curriculum needs, institutional policies, and available informatics support.

Architecture and safeguards

The LLM was ChatGPT-4o3 “Deep Research” (OpenAI, San Francisco, CA), used as a tool for literature retrieval and hypothesis generation rather than for diagnostic adjudication [11]. Each interaction followed the fixed structure described above to reduce hallucination and enhance traceability. Chain-of-thought outputs were preserved only as reasoning traces for coaching and error analysis [13,14].

Rigor and safety controls paralleled our pedagogical aims [9,10]. Only de-identified inputs were used. Candidate predictors advanced to the variable dictionary only when they were pathophysiologically plausible and recurrent across independent studies [4,5]. These internal safeguards were aligned with external guidance emphasizing transparency, human oversight, and documentation in health-related AI systems, including the CONSORT-AI extension, DECIDE-AI guideline, and MI-CLAIM checklist [3,6-8]. The overall analytical pathway was documented so that future evaluations can map the workflow onto emerging AI reporting standards, including CONSORT-AI, DECIDE-AI, and the MI-CLAIM checklist [6-8].

Alignment with AI reporting standards

The workflow was intentionally designed to mirror key domains in the CONSORT-AI extension, the DECIDE-AI, and the MI-CLAIM checklist [6-8]. Case reconstruction and de-identification address data provenance and input specification; the prompt ledger and archived model identifiers document human-AI interactions and analytic procedures; and the variable dictionary with Neurologic Deterioration in Brain Abscess Score (NDBAS v0.1) tiers makes decision rules explicit and reproducible.

NDBAS derivation and index-case result

The provisional Neurologic Deterioration in Brain Abscess Score (NDBAS v0.1) schema and its application to the index case are summarized in Table 4.

**Table 4:** NDBAS v0.1 schema with index-case mapping and total score. This table summarizes the NDBAS v0.1 components and thresholds and maps the index case to each component with check marks and point totals. Each component is scored 0–2 based on predefined categories; the index-case value and awarded points are shown in the final columns. Full operational definitions are detailed in the variable dictionary (Table 3). Variables and conservative 0–2 weights emphasize mass-effect (diameter, midline shift, edema) and sensorium (GCS); laboratory activity and comorbidity act as modifiers. The index-case values (bilobed ~2.6 cm, mild shift, moderate edema, GCS = 15, leukocytosis, diabetes) sum to NDBAS = 5 (moderate risk). Thresholds for diameter and shift follow severity/index literature and clinical syntheses [4,5]. Footnote: * Source/pathogen clues (e.g., odontogenic focus, *Streptococcus anginosus* group) are treated as risk-raising context and encoded as modifiers in the variable dictionary; they did not contribute primary points in NDBAS v0.1. Abbreviations: GCS, Glasgow Coma Scale; NDBAS, Neurologic Deterioration in Brain Abscess Score; CRP, C‑reactive protein; SAG, Streptococcus anginosus group.

NDBAS component	0-point category	1-point category	2-point category	Index-case value	Points awarded
Glasgow Coma Scale (sensorium/neuro examination)	15	13–14	≤12	15	0
Abscess diameter (imaging)	<2.5 cm	2.5–3.0 cm	>3.0 cm	2.6 cm	1
Midline shift (imaging)	None	1–4 mm	≥5 mm	~2 mm	1
Perilesional edema (imaging)	None/mild	Moderate	Severe	Moderate	1
Number/location (imaging)	Single, non-eloquent region	Multiple or eloquent regions	Posterior fossa plus multiple lesions	Single, frontoparietal	0
Inflammatory activity (laboratories)	Absent or stable	Leukocytosis or rising CRP	Escalating on therapy	Leukocytosis	1
Comorbidity (history)	None	Diabetes or vascular risk	Immunosuppression	Diabetes	1
Source/pathogen clue (history/laboratories)	None or uncertain	Odontogenic source or SAG group	Not defined in v0.1	Poor dentition; SAG likely	0*

**Figure 1:**
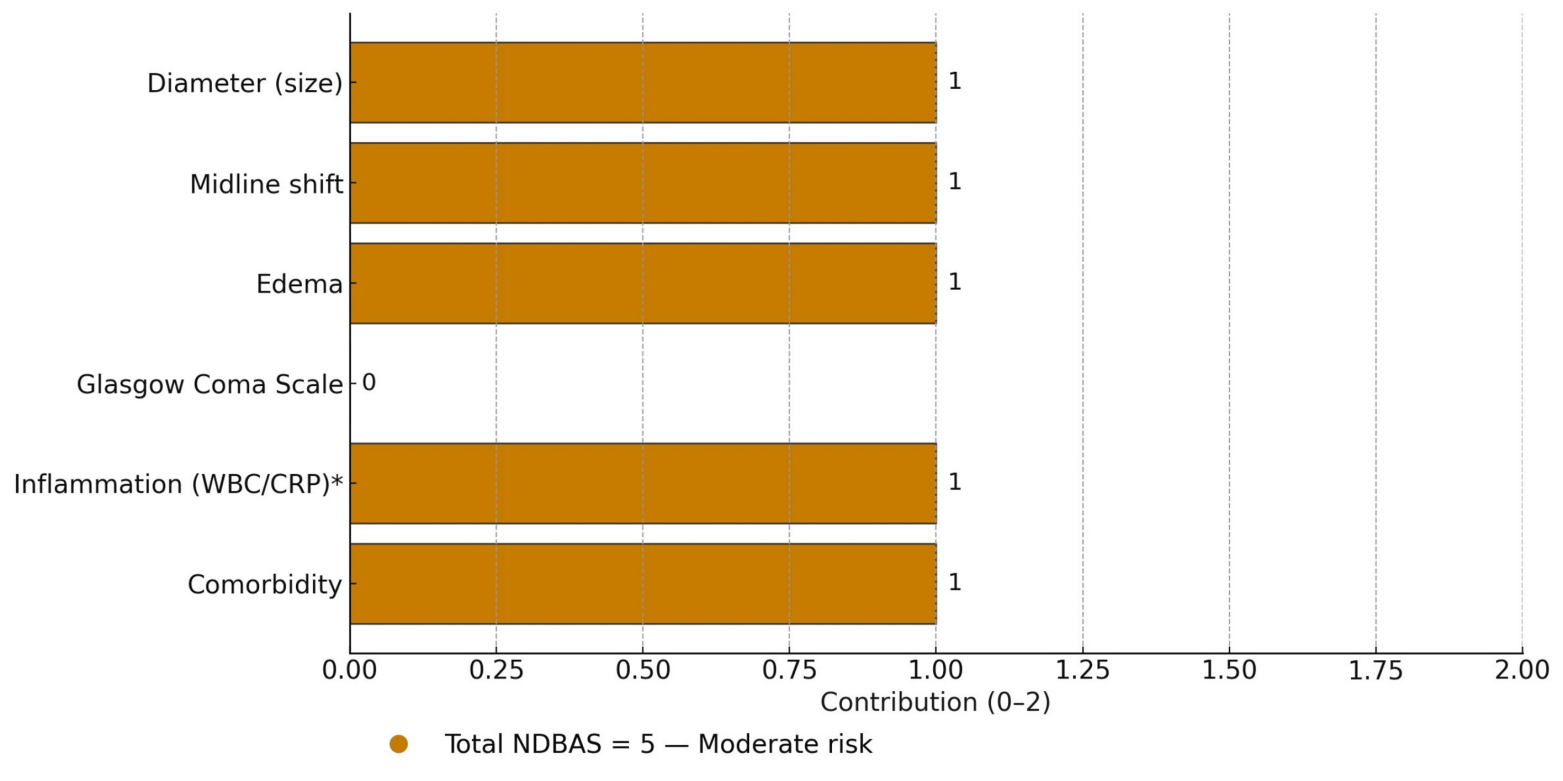
Bar chart of NDBAS v0.1 — index-case score breakdown by variable. NDBAS breakdown bar chart shows five variables contributing one point each (diameter, midline shift, edema, inflammation, comorbidity) and GCS contributing 0; total score 5 = moderate risk. Horizontal bars show the 0–2 contribution from each component (diameter, midline shift, edema, Glasgow Coma Scale, inflammation, comorbidity); total NDBAS = 5 indicates moderate risk. * Inflammation (WBC/CRP) is treated as a modifier pending cohort validation. Figure 1 (NDBAS v0.1 breakdown bar chart) was generated by the authors using Python/matplotlib, with a layout variant rendered using an OpenAI generative image service under direct human direction. Abbreviations: GCS, Glasgow Coma Scale; WBC, white blood cell count; CRP, C‑reactive protein; NDBAS, Neurologic Deterioration in Brain Abscess Score.

The score comprises variables that emphasize mass effect and sensorium, with inflammatory activity and comorbidity functioning as modifiers. When the schema was applied to the de-identified index patient, the total score was five, corresponding to a moderate-risk tier within this educational framework. This numeric value is not intended to convey predictive performance; it functions only as a structured way to organize deterioration anchors for teaching and reflection. The diameter and midline-shift thresholds used in NDBAS v0.1 follow radiology-based severity constructs and clinical syntheses from prior brain abscess literature [4,5]. Figure 1 provides a bar-chart visualization of this same case-level breakdown, serving as a teaching aid to illustrate how each domain contributes to the overall conceptual score.

**Figure 2:**
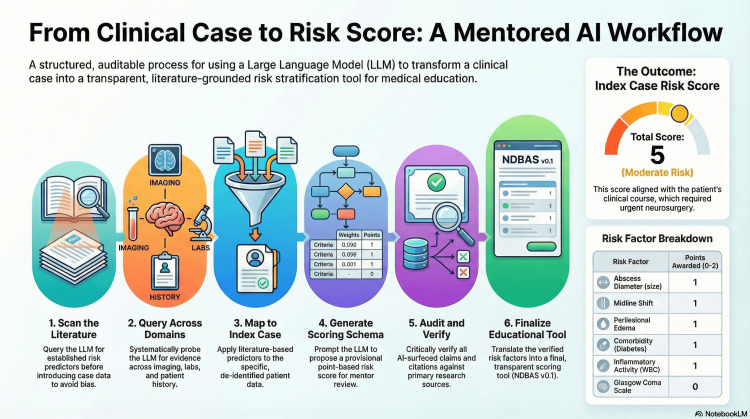
From clinical case to AI-powered insight: A mentored workflow for medical education. This infographic of the AI-assisted risk-stratification workflow depicts the progression from de-identified case reconstruction, through domain-scoped prompting and the ask–verify–revise cycle, to the creation of the prompt ledger, variable dictionary, and final NDBAS v0.1 schema. The steps 1-5 are detailed in the Methods, with initial step 1: "Scan the Literature" to build up the prior knowledge on the current understanding of pathophysiology of infective brain abscess in adults with or without comorbidities. This step frames the established risk predictors before introducing index case data to avoid serious biases. The infographic was created using NotebookLM based on the authors’ de-identified case summary and variable dictionary, again under direct author oversight.

This reinforces the score’s conceptual design, in which mass-effect variables and neurologic status anchor the core score and systemic factors conservatively adjust risk upward. The overall analytic trajectory, from case reconstruction to LLM-assisted synthesis, score generation, and educational deployment, is summarized in an infographic presented in Figure 2.

This figure situates NDBAS derivation within the ask-verify-revise cycle, highlighting de-identification, documentation, and human oversight, and visually aligning the workflow with contemporary AI governance frameworks that stress rigor, reproducibility, and transparency in health-related AI [3,6-8].

Relative to a purely manual scoping review and codebook build, the mentored LLM-assisted synthesis reduced time spent on evidence gathering and variable operationalization. Based on the student’s prior experience with manual methods, the ask-verify-revise cycle shortened the process by an estimated 40 hours, primarily by accelerating identification of candidate variables, thresholds, and primary-source anchors for mentor adjudication. This efficiency gain is particularly relevant for pre-residency scholarship, where trainees balance research with clinical duties and exam preparation, and it aligns with prior work showing that LLMs can encode and rapidly surface clinically relevant knowledge [11] and support structured trainee-led risk-stratification projects [12]. Even with time savings, rigor was preserved through primary-source verification, pathophysiologic checks, and mentor approval before any variable entered the final schema, reflecting the rigor, reproducibility, and transparency principles emphasized in AI reporting checklists [6-8].

## Discussion

In this single-case educational project, the provisional Neurologic Deterioration in Brain Abscess Score (NDBAS v0.1) classified the index patient as moderate risk (total score of 5), a result that was concordant with his clinical course culminating in urgent neurosurgical drainage. The principal contributors to the score, including lesion size, midline shift, edema, preserved sensorium, inflammatory activity, and comorbidity, mirror severity signals already emphasized in the literature, particularly the radiologic severity constructs of Demir et al. and the clinical syntheses of Brouwer et al. [4,5]. We do not attribute any prognostic power to this numeric value; rather, the score is used as an educational scaffold to make deterioration anchors explicit, discussable, and auditable. Instead of claiming predictive performance based on a single case, our intent is to show that a supervised LLM-assisted workflow can recover and operationalize these established deterioration signals in a transparent and reproducible way, suitable for pre-residency scholarship.

Educationally, the mentored LLM process functioned as a transparency aid and research assistant rather than an oracle. The prompt ledger and chain-of-thought traces created an auditable substrate for coaching, and the need for human oversight was concrete rather than theoretical. For example, in iteration 4 of the ledger (Table 2), the model proposed a weighting scheme that appeared internally plausible but was opaque in its justification. The mentor rejected this structure, recorded the decision as “Modify,” and redirected the student toward a conservative 0-2 scale anchored to clearly documented cut points and primary-source evidence from brain abscess cohorts [4,5]. This illustrates how the ledger operates as an active brake on AI-suggested structure: it records when the model’s contribution is helpful, incomplete, or incorrect, and it keeps chain-of-thought outputs in their proper role as reasoning traces that require independent verification. This pattern aligns with the broader view that LLMs should support hypothesis generation within documented, human-in-the-loop workflows guided by rigor, reproducibility, and transparency standards [3,6-8,11,13,14].

The observed efficiency gains must be interpreted through this educational lens. Based on contemporaneous session notes, the ask-verify-revise cycle reduced scoping and codebook-construction time by roughly one workweek (about 40 person-hours) compared with the student’s prior manual approach. Crucially, that time was not simply “saved” but reallocated. Instead of repetitive database searches and ad hoc notetaking, effort shifted toward higher-value activities: checking citations against PubMed-indexed studies, debating the plausibility of proposed predictors, and negotiating operational definitions. Decisions such as treating a 5-mm midline shift as the highest-risk tier or encoding leukocytosis and rising C-reactive protein as modifiers rather than primary drivers required careful reading and discussion grounded in existing evidence [4,5]. In this sense, the LLM displaced busy work while amplifying opportunities for critical appraisal, an attractive trade-off in pre-residency settings where time is limited and scholarly expectations are increasing, and consistent with prior work showing that LLMs can encode clinically relevant knowledge and support student-led risk-stratification projects [11,12].

From an educational standpoint, this differs in important ways from a traditional case report or a conventional student-led scoping review. In a standard case report, the learner typically produces a linear narrative with a brief literature summary; the intermediate reasoning, search strategy, and variable operationalization often remain invisible. Likewise, many student scoping projects leave only scattered search histories or informal notes. In contrast, the prompt ledger and variable dictionary capture the full analytic pathway in a structured, machine-readable form, making the steps of question framing, evidence appraisal, threshold selection, and model refinement directly observable for teaching and feedback, consistent with cognitive apprenticeship and self-regulated learning frameworks [9,10].

For learners who lack the pattern-recognition skills of experienced clinicians, the workflow also provides a scaffolded entry into genuine scholarship. The student did not simply receive a finished score; he co-constructed the prompt ledger and variable dictionary (Tables 2, 3), translating vague notions such as “mild edema” or “worsening neurologic status” into explicit, quantifiable variables. Building NDBAS v0.1 required each deterioration signal, including mass effect, sensorium, inflammatory activity, comorbidity, and source clues, to be defined, encoded, and linked to specific evidence anchors [4,5]. This process of operationalization is central to academic residency and clinical research but is rarely made visible in traditional case reports. Here, the ledger and dictionary become durable academic products and teaching tools: they allow mentors to see how students frame questions, respond to ambiguous evidence, and learn to justify thresholds rather than accept them as givens, in line with cognitive apprenticeship and self-regulated learning frameworks [9,10].

Limitations

This technical report has several important limitations. First, it is based on a single de-identified case and yields a conceptual, education-focused model rather than a validated clinical decision tool; we make no claims about discrimination, calibration, or net clinical benefit, and the Neurologic Deterioration in Brain Abscess Score (NDBAS v0.1) is explicitly framed as an educational scaffold [4,5]. Second, several components, particularly inflammatory modifiers such as C-reactive protein trajectories, rest on heterogeneous or sparse evidence and were therefore encoded as low-weight modifiers rather than core drivers [4]. Third, the LLM is constrained by the published record on which it was trained and is more likely to highlight text-rich variables such as lesion diameter and midline shift than subtler visual patterns that may be underreported or difficult to capture in prose; moreover, the model never accessed raw imaging, only clinician-generated reports, further narrowing its representational space [4,5,11]. Finally, the estimated 40-hour reduction in effort reflects comparison with a single student’s prior manual workflow and is not based on a formal time-and-motion study. Safeguards such as strict de-identification, primary-source verification, pathophysiologic plausibility checks, and mentor review mitigate, but do not eliminate, risks of bias, omission, or hallucination [3,6-8,11,13,14].

Future work and educational implications

Future work should move beyond this single-case prototype. A prospective, multi-case program is needed to refine thresholds and weights, assess inter-rater reliability for learner-extracted variables, and examine how consistently different students populate the prompt ledger and variable dictionary when given the same case. Educational outcomes also merit formal evaluation, including gains in prompt literacy, comfort with artificial intelligence governance concepts such as rigor, reproducibility, and transparency, and improvements in the visibility of reasoning as judged by faculty [2,3,6-8]. Integration into formal curricula will require alignment with institutional AI policies and established reporting frameworks such as the CONSORT-AI extension, the DECIDE-AI, and the MI-CLAIM checklist, so that any eventual movement toward clinical application proceeds under clear standards [6-8]. Because this workflow was piloted with a single fourth-year medical student working closely with one faculty mentor, and the estimated 40-hour efficiency gain is based on local comparison rather than a formal time-and-motion study, both the educational and efficiency signals should be interpreted as hypothesis-generating rather than definitive [3,6-10,12-14].

Within these boundaries, mentored LLM use is best positioned not as an autonomous diagnostician but as an accelerator for literature synthesis and hypothesis formation, precisely the zone where artificial intelligence can add value to pre-residency scholarship without displacing human judgment [1,3,11,12]. By converting ephemeral chatbot exchanges into stable, auditable artifacts, the prompt ledger, variable dictionary, and conceptual Neurologic Deterioration in Brain Abscess Score (NDBAS v0.1), this workflow offers a practical, replicable pattern for bringing AI into medical student research in a way that is transparent, supervised, and educationally rich [3,6-10,12-14].

## Conclusions

This technical report illustrates a practical route to building pre-residency artificial intelligence literacy by embedding a structured, audit-ready LLM workflow into complex cases. Starting from a de-identified brain abscess narrative, a supervised ask-verify-revise process moved the learner from casual chatbot use to transparent evidence synthesis: posing focused clinical questions, tracing model-assisted reasoning, and defending each variable with primary literature. The prompt ledger was central to this shift, converting fleeting AI exchanges into a durable audit trail that faculty can review, teach from, and assess. The resulting Neurologic Deterioration in Brain Abscess Score (NDBAS v0.1) mapped established deterioration anchors, i.e., lesion size, midline shift, sensorium, inflammatory activity, and comorbidity, onto a single case in a reproducible way and is explicitly positioned as an educational prototype rather than a clinical decision tool, with no intended predictive or triage function at the bedside.

Equally important, the workflow redistributed the learner’s effort from low-yield manual scoping toward higher-order activities such as checking citations, debating thresholds, and refining cut points. In doing so, it operationalized cognitive apprenticeship in a concrete pattern where students learn to treat LLMs as transparency aids rather than oracles. For educators and curriculum leaders, the key implication is that this pattern, i.e., prompt ledger plus ask-verify-revise, can be adapted to other complex cases and disease domains with modest effort, generating parallel curriculum artifacts such as narrative case appendices, prompt ledgers, and variable dictionaries. With appropriate governance, de-identification, and faculty oversight, similar mentored workflows can help students turn single cases into auditable conceptual models, strengthening both their scholarly output and their readiness to engage with AI responsibly during clinical training.

## Appendices

**Table 5:** Acute right-sided weakness and dysarthria in a 60-year-old male. De-identified, structured timeline of the brain abscess case, summarizing history, examination, imaging, laboratory data, interventions, and severity trajectory in the same time-stamped format used for the Neurologic Deterioration in Brain Abscess Score (NDBAS v0.1) derivation. Abbreviations: BP, blood pressure; HR, heart rate; RR, respiratory rate; SpO_2_, oxygen saturation; RA, room air.

Timepoint/phase	History/key events	Neurologic & physical findings	Imaging	Laboratory & microbiology	Interventions/consultations	Clinical trajectory/severity
ED presentation (initial stroke alert)	A 60-year-old male with a history of hypertension, type 2 diabetes mellitus, chronic obstructive pulmonary disease, and coronary artery disease with prior stents awakens with sudden right arm and leg weakness, slurred speech, and left facial droop. Mild diffuse headache; denies trauma, seizure, fever, chest pain, or dyspnea. Reports adherence to medications. Lives alone with limited social support.	Vitals: BP = 158/88 mm Hg, HR = 70 bpm, RR = 12, temperature = 36.7°C, SpO₂ = 97% on RA. Awake, alert, oriented ×3. Dysarthric. Left facial droop. Right hemiplegia; left-sided strength = 5/5. No meningismus. Poor dentition with multiple decayed molars and periodontal disease.	Non-contrast CT head: Left frontal–parietal edema with ~1.8 cm lesion; no acute hemorrhage. Findings nonspecific (possible underlying lesion vs. acute ischemia).	Initial labs show leukocytosis, consistent with systemic inflammation (other chemistries without critical derangements).	Code stroke activated. Emergent CT performed. The stroke team was consulted. Began stroke workup; no thrombolysis given due to presentation outside tissue plasminogen activator (tPA) window.	High suspicion for acute cerebrovascular accident (stroke mimic not yet recognized). Neurologic deficits are significant but hemodynamically stable; early concern for mass lesion vs. ischemia.
Early in-hospital workup (diagnostic clarification)	Ongoing focal deficits and mild headache; no new systemic complaints.	Persistent right-sided hemiparesis and dysarthria; left facial droop; overall stable mental status.	MRI brain with and without contrast: Bilobed rim-enhancing mass in the left frontoparietal region, up to 2.6 cm, with surrounding vasogenic edema and ~2 mm rightward midline shift; intense internal restricted diffusion. Mild chronic microvascular ischemic disease. CT soft tissues of head/neck: Advanced dental and periodontal disease, no discrete fluid collection. Transesophageal echocardiogram: No vegetations or evidence of endocarditis.	Leukocytosis persists. Metabolic panel otherwise without critical derangements.	Diagnostic focus shifts from ischemic stroke to mass lesion (cerebritis/abscess vs. tumor). Neurosurgery and infectious diseases were consulted. Corticosteroids were initiated for cerebral edema. Seizure precautions implemented. Broad-spectrum intravenous antibiotics (β-lactam/β-lactamase inhibitor) started empirically.	Pattern of a rim-enhancing lesion with restricted diffusion, edema, and mild midline shift in the setting of poor dentition raises a great concern for a brain abscess with early mass effect. Severity is considered moderate but potentially unstable if mass effect progresses.
Operative management (craniotomy and drainage)	Indication for surgery based on lesion size, edema, and evolving mass effect in a comorbid patient.	Preoperative neurologic exam continues to show significant right-sided weakness and dysarthria; mental status preserved.	Preoperative imaging used for operative planning; findings unchanged in character (bilobed rim-enhancing lesion with edema and shift).	Routine preoperative labs monitored; leukocytosis and inflammatory profile guide ongoing antibiotic therapy.	Left frontoparietal craniotomy with resection of the mass was performed. Intraoperative findings reveal a purulent collection consistent with an abscess rather than a solid tumor. Specimens sent for microbiologic analysis.	Surgical decompression directly addresses mass effect and allows definitive diagnosis. Short-term severity is high (neurosurgical procedure), but the procedure aimed at preventing deterioration from expanding abscess and increased intracranial pressure.
Early postoperative course (ICU/monitored setting)	The postoperative period focused on stabilization and management of cerebral edema and infection.	Gradual improvement in neurological status over hospital stay; persistent but improving right-sided deficits and dysarthria.	Postoperative imaging (where obtained) shows improvement in mass effect and reduction of midline shift after evacuation of the abscess.	Ongoing monitoring of leukocytosis and inflammatory markers; metabolic panel followed for hyponatremia and other complications. Cultures from abscess: growth of Streptococcus intermedius (Streptococcus anginosus group).	Continued hyperosmolar therapy, including hypertonic saline; tolvaptan used to manage hyponatremia and cerebral edema. Broad-spectrum IV antibiotics continued via peripherally inserted central catheter (PICC) and tailored to S. intermedius per infectious diseases recommendations. Multidisciplinary management by neurosurgery, infectious diseases, pharmacy, nutrition, nephrology, and rehabilitation services.	Mass effect and midline shift improve with combined surgical and medical therapy. Identification of S. intermedius plus severe dental disease supports an odontogenic source. Clinical trajectory shifts from high risk of deterioration to cautiously improving but still vulnerable postoperative state.
Source identification and ongoing management	Historical context of poor dentition and longstanding diabetes revisited as probable contributors to abscess formation.	Neurologic deficits continue to improve with rehabilitation, though not yet at baseline.	No new intracranial lesions identified; postoperative course focused on resolution of edema and prevention of recurrence.	Microbiologic confirmation of S. intermedius; no evidence of endocarditis on prior transesophageal echocardiogram; inflammatory markers trended to monitor response.	The oral and maxillofacial surgery team was consulted for the extraction of diseased molars once clinically stable to achieve source control. Prolonged IV antibiotic course maintained with close monitoring.	Etiology was established as an odontogenic brain abscess in a high-risk host (diabetes, vascular disease, poor dentition). Severity reclassified from acute neurologic emergency to subacute recovery phase with ongoing risk if source not fully controlled.
Discharge planning and follow-up (rehabilitation phase)	Transition planning focuses on functional recovery and long-term infection control.	Residual motor and speech deficits present but improved; patient participates in therapies and is medically stable for transfer.	Imaging demonstrates stable or improving postoperative changes with no new abscess formation.	Laboratory parameters, including leukocytosis and metabolic markers, show normalization or clear improvement under therapy.	Patient discharged to an inpatient rehabilitation facility for intensive physical, occupational, and speech therapy. Follow-up arranged with neurosurgery, infectious diseases, and dental/oral surgery for definitive management of oral pathology and completion of IV antibiotics.	Acute intracranial risk substantially reduced; patient enters recovery and rehabilitation phase. Long-term outcomes will depend on neurologic recovery, adherence to the antimicrobial regimen, and success of dental source control.
